# Interplay Between Age and Neuroinflammation in Multiple Sclerosis: Effects on Motor and Cognitive Functions

**DOI:** 10.3389/fnagi.2018.00238

**Published:** 2018-08-08

**Authors:** Alessandra Musella, Antonietta Gentile, Francesca Romana Rizzo, Francesca De Vito, Diego Fresegna, Silvia Bullitta, Valentina Vanni, Livia Guadalupi, Mario Stampanoni Bassi, Fabio Buttari, Diego Centonze, Georgia Mandolesi

**Affiliations:** ^1^Laboratory of Synaptic Immunopathology, IRCCS San Raffaele Pisana, Rome, Italy; ^2^San Raffaele University of Rome, Rome, Italy; ^3^Laboratory of Synaptic Immunopathology, Department of Systems Medicine, University of Rome Tor Vergata, Rome, Italy; ^4^Unit of Neurology, Istituto Neurologico Mediterraneo (IRCCS Neuromed), Pozzilli, Italy

**Keywords:** aging, cognition, experimental autoimmune encephalomyelitis, multiple sclerosis, neurodegeneration, neuroinflammation, synaptic plasticity, synaptopathy

## Abstract

Aging is one of the main risk factors for the development of many neurodegenerative diseases. Emerging evidence has acknowledged neuroinflammation as potential trigger of the functional changes occurring during normal and pathological aging. Two main determinants have been recognized to cogently contribute to neuroinflammation in the aging brain, i.e., the systemic chronic low-grade inflammation and the decline in the regulation of adaptive and innate immune systems (immunosenescence, ISC). The persistence of the inflammatory status in the brain in turn may cause synaptopathy and synaptic plasticity impairments that underlie both motor and cognitive dysfunctions. Interestingly, such inflammation-dependent synaptic dysfunctions have been recently involved in the pathophysiology of multiple sclerosis (MS). MS is an autoimmune neurodegenerative disease, typically affecting young adults that cause an early and progressive deterioration of both cognitive and motor functions. Of note, recent controlled studies have clearly shown that age at onset modifies prognosis and exerts a significant effect on presenting phenotype, suggesting that aging is a significant factor associated to the clinical course of MS. Moreover, some lines of evidence point to the different impact of age on motor disability and cognitive deficits, being the former most affected than the latter. The precise contribution of aging-related factors to MS neurological disability and the underlying molecular and cellular mechanisms are still unclear. In the present review article, we first emphasize the importance of the neuroinflammatory dependent mechanisms, such as synaptopathy and synaptic plasticity impairments, suggesting their potential exacerbation or acceleration with advancing age in the MS disease. Lastly, we provide an overview of clinical and experimental studies highlighting the different impact of age on motor disability and cognitive decline in MS, raising challenging questions on the putative age-related mechanisms involved.

## Introduction

Multiple sclerosis (MS) is an inflammatory neurodegenerative disease of the central nervous system (CNS), mainly affecting young adults. Its exact etiology remains unknown, but it is widely accepted that genetically susceptible individuals develop MS after exposure to undefined environmental triggers. MS is primarily characterized by a breakdown in immune tolerance to myelin and neuronal antigens and by a dysfunction of the blood brain barrier (Gourraud et al., 2012; Beecham et al., 2013; Olsson et al., 2017). Typically, infiltrating myelin-reactive lymphocytes (mainly T-cells but also B-cells) attack myelin sheaths and axon antigens on oligodendrocytes and neurons in the CNS. This event causes an inflammatory cascade, formation of large demyelinating plaques in the white matter and gliosis, neuroaxonal degeneration and synaptopathy, leading to an impairment of the neuronal signaling and, later on, to neurodegeneration (Compston and Coles, 2008; Dendrou et al., 2015; Mandolesi et al., 2015). Clinical manifestations include motor impairments, sensory and visual disturbances, fatigue, pain, mood disturbances and cognitive deficits, in relation to the spatiotemporal dissemination of pathological lesion sites in the CNS (Dendrou et al., 2015). Most of MS patients start with a relapsing remitting phase (RRMS), which later develops into a secondary progressive phase (SPMS). In primary progressive MS (PPMS) patients, the relapsing stage is absent and the disease starts already with a progressive loss of neurological functions (Compston and Coles, 2008; Disanto et al., 2010; Lassmann et al., 2012). Innate reparative processes can occur through remyelinating and neuronal repairing processes, which are highly variable among patients (Stangel, 2008; Bramow et al., 2010).

An important feature of adult MS is chronological age, consisting in a defined age interval between the mid-twenties and late-thirties for disease onset and a time limit, after the fifth decade, when the disorder is rarely diagnosed (Polliack et al., 2001; Sanai et al., 2016). Mounting evidence suggests that prognosis of MS appears to be, at least to some extent, dependent of age and not markedly influenced by the initial, exacerbating-remitting or progressive disease course (Confavreux and Vukusic, 2006; Scalfari et al., 2011; Cossburn et al., 2012; Sanai et al., 2016; Roy et al., 2017; Ruano et al., 2017). Many age-related changes affecting the brain could collectively affect neuronal viability and vulnerability in MS: increased iron accumulation, oxidative stress followed by mitochondrial injury, decrease of trophic support from the peri-plaque environment, decline of remyelination, chronic, systemic low grade inflammation as well as a broad increase in the production of inflammatory molecules such as pro-inflammatory cytokines (“inflammaging”; Pizza et al., 2011; Dorszewska, 2013; Dendrou et al., 2015; Di Benedetto et al., 2017; Bolton and Smith, 2018). Although further efforts are necessary to better clarify this aspect, the present review highlights common inflammatory processes occurring in both aging and MS brain that might be critical for understanding why age is an important risk factor for MS disability (motor and cognitive) and progression.

## Immune System Dysregulation in MS: The Impact of Aging

Key age-associated changes in the CNS are triggered by microglia, their impaired regulation (low-grade inflammation and inflammaging) and the inflammatory and oxidative stressful environment they build up. Immune challenges, such as infections, surgery, or traumatic brain injuries, result in greater susceptibility to memory impairments and altered synaptic plasticity during aging (Ojo et al., 2015; Cornejo and von Bernhardi, 2016; Matt and Johnson, 2016; Bettio et al., 2017; Di Benedetto et al., 2017). On the other hand, advanced age is associated with a phenomenon called immunosenescence (ISC) which refers to a weakening in integrity and efficiency of the adaptive and innate immune systems (Walford, 1969; Denkinger et al., 2015; Di Benedetto et al., 2017).

**Figure 1:**
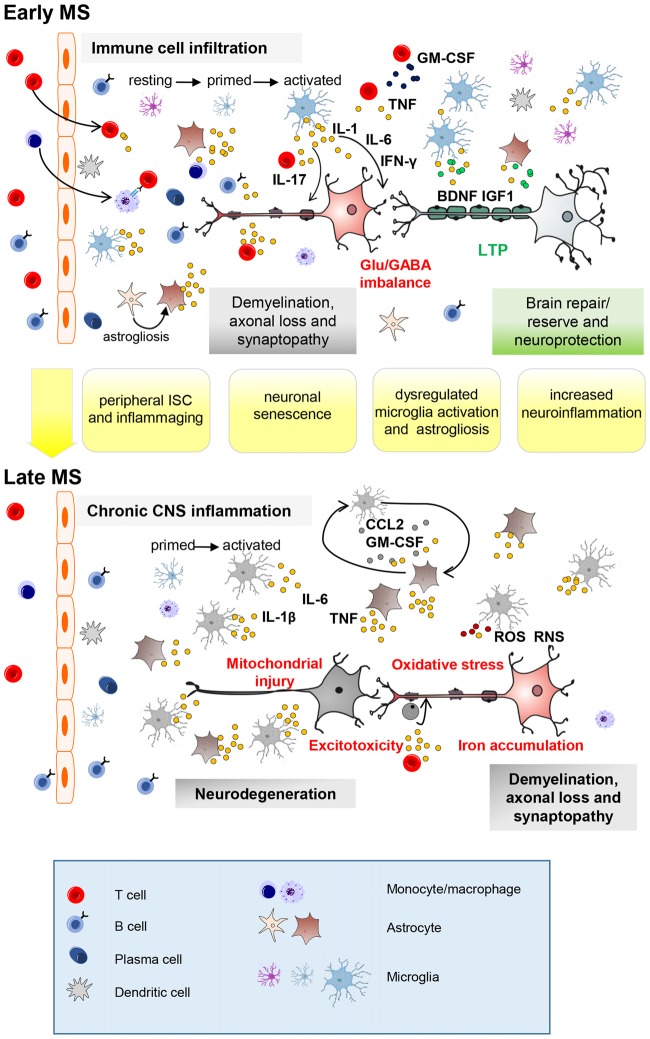
Key neuroinflammatory processes occurring in the central nervous system (CNS) of multiple sclerosis (MS) patients influenced by aging. Immune cell infiltration from the periphery, mainly through the blood–brain barrier, is a prominent feature of early-stage MS (top panel). Peripheral innate and adaptive immune cells, along with activated CNS-resident microglia and astrocytes, promote demyelination, axonal injury and synaptopathy mainly through action of soluble inflammatory (IL-1β, Interleukin-1β; TNF, tumor necrosis factor; IFN-γ, interferon-γ; IL-17, interleukin-17) and neurotoxic mediators. Neuronal damage can be completely or partly resolved due to remyelination, resolution of inflammation and neuroprotective/reparative mechanisms (growth factors). Furthermore, additional mechanisms, from local synaptic plasticity to brain rewiring, intervene to functionally compensate synaptic loss (long-term potentiation, LTP). With advancing years, premature aging processes such as peripheral immunosenescence (ISC) and inflammaging may lead to age-related changes in the blood. Neuronal senescence and unusual microglia (primed) activation as well as astrogliosis (IL-1β; IL-6; TNF; granulocyte–macrophage colony-stimulating factor, GM-CSF; CC-chemokine ligand 2, CCL2) might contribute at exacerbating MS neuroinflammatory processes (synaptopathy, impaired synaptic plasticity, reduced production of brain derived neurotrophic factor (BDNF) and insuline-like growth factor (IGF-1)). Later on (bottom panel), immune cell infiltration wanes, perhaps due to adaptive immune cell exhaustion from chronic antigen exposure. However, chronic CNS-intrinsic inflammation, and other processes influenced by age (such as oxidative stress, mitochondrial injury, iron accumulation and excitotoxicity) might contribute at exacerbating early pathological processes leading to neurodegeneration. Further microglial recruitment and activation might be induced by interaction with astrocytes releasing CCL2 and GM-CSF as occur during aging. Astrocytes can prevent remyelination at sites of neuroaxonal injury by inhibiting progenitor cells from developing into mature oligodendrocyte cells (ODCs). RNS, reactive nitrogen species; ROS, reactive oxygen species.

In past years, researchers have spent much effort to understand the impact of aging and ISC on adaptive immunity in MS and in its rodent model, experimental autoimmune encephalomyelitis (EAE). From these studies, which were primarily focused on the analysis of biomarkers of ISC at peripheral level (Bolton and Smith, 2018), emerged that a premature ISC might operate during the course of MS and of EAE (Bolton and Smith, 2018). The effects of a prematurely aged immune system and ISC on MS and EAE are still unknown, but emerging data justifies and encourages further investigation. ISC typically affects both adaptive and innate immune systems. The most relevant changes in the adaptive immunity are decreased peripheral naïve T cells and concomitant accumulation of late-stage differentiated memory T cells, with reduced antigen receptor repertoire diversity. This phenomenon results from age-related impairments in the hematopoietic stem cell compartment (which generates few T-cell precursors), and from thymic involution. An increase of circulating cytokines facilitates a systemic and chronic, low-grade, inflammatory state which is typical of aging (termed “inflammaging”). This systemic inflammation may promote neuroinflammation by modulating glial cells, leading to an increased risk for developing neurodegeneration and cognitive impairment (CIs) in healthy individuals (Pizza et al., 2011; von Bernhardi et al., 2015; Di Benedetto et al., 2017). In MS patients, the additional inflammation carried by peripheral immune cell infiltration and by CNS-resident immune cell activation may significantly accelerate the inexorable aging processes in the CNS, and as the resulting stress response is excessive for homeostasis to be properly preserved (Dendrou et al., 2015), prominent neurodegenerative processes progressively follow (Figure 1).

Accumulating evidence suggests that an exacerbation of common neuropathological aspects of MS and of aging brain, such as synaptic dysfunction and loss (synaptopathy) and synaptic plasticity impairment (Di Filippo et al., 2008; Weiss et al., 2014; Mandolesi et al., 2015; Stampanoni Bassi et al., 2017), might contribute to explain why aging may impact on MS disability.

## Inflammatory Synaptopathy and Synaptic Plasticity in MS/EAE and in Aging Brain

### Synaptopathy

Synaptophaty refers to a progressive dysfunction and loss of the synaptic compartment of the CNS that is emerging as a pathophysiological feature of MS disease and of its mouse model EAE (Mandolesi et al., 2015; Henstridge et al., 2016). Along with demyelination and axonal damage, synaptopathy contributes to the neurodegenerative decline of the CNS starting since the early phase of the disease. In MS and EAE, neuroinflammation is responsible for an imbalance between the glutamatergic and GABAergic systems in the brain and spinal cord. During acute inflammatory attacks in MS and EAE, pro-inflammatory cytokines (such as tumor necrosis factor (TNF); interleukin-1β (IL-1β)), released from activated microglia and astroglia as well as from infiltrating lymphocytes, trigger a progressive increase of the glutamatergic transmission and an impairment of the GABAergic synaptic response, leading to uncontrolled excitability and possibly to neurodegeneration (Centonze et al., 2009; Rossi et al., 2011, 2012, 2014; Mandolesi et al., 2012, 2013, 2017a; Nisticò et al., 2013; Mori et al., 2014a). Synaptopathy has long-lasting effects (such as excitotoxic damage) that can give rise to motor and CIs. It is remarkable that traits of neuroinflammatory synaptopathy are evident also in normal aging brains (Viviani and Boraso, 2011; Barrientos et al., 2015; Bettio et al., 2017), strongly suggesting an exacerbation with age of pathological events occurring in MS (Figure 1). It has been indeed suggested that during aging, peripheral ISC and inflammaging may favor the activation of microglia cells, leading to loss of their neuroprotective functions, to neuronal dysfunctions and tissue damage (Henry et al., 2009; Norden and Godbout, 2013; Matt and Johnson, 2016). The over-production of pro-inflammatory mediators, such as IL-1β, interleukin-6 (IL-6) and TNF, disrupts the delicate balance needed for synaptic homeostasis by modulating ion channels and glutamate receptors (Viviani and Boraso, 2011). In particular, data mainly obtained in animal models suggest that IL-1β and TNF exacerbate or favor excitotoxicity by modulating the N-methyl-D-aspartate (NMDA) and α-amino-3-hydroxy-5-methyl-4-isoxazole propionic acid (AMPA) receptors in the aging brain, mimicking the alterations occurring in MS and EAE brains (Jasek and Griffith, 1998; Viviani et al., 2003, 2006; Stellwagen et al., 2005; Centonze et al., 2009; Viviani and Boraso, 2011; Grasselli et al., 2013). Inflammatory dependent glutamate excitotoxicity and reduced production of neurotrophic factors (brain derived neurotrophic factor, BDNF; insuline-like growth factor, IGF-1) may impact neuronal functions (Figure 1) in aging brain (von Bernhardi et al., 2010; Dorszewska, 2013; Di Benedetto et al., 2017).

### Synaptic Plasticity

Compelling evidence suggests that during aging, neuroinflammation may contribute to impaired long-term potentiation (LTP), which has been classically regarded, together with long-term depression (LTD), as the substrate for learning and memory processes (Viviani and Boraso, 2011; Barrientos et al., 2015; Di Benedetto et al., 2017).

The aged brain seems to be remarkably sensitive to an immune challenge in the periphery due to an unusual hyperactivation of microglia cells that, in cooperation with impairments in key regulatory systems, causes a long lasting neuroinflammatory state and age-related neurobehavioral complications (Norden et al., 2015; Di Benedetto et al., 2017). Age-related cognitive decline following an immune challenge likely depends on an exaggerated inflammatory response. In particular it has been shown in rodents that the pro-inflammatory cytokines IL-1β can impair synaptic plasticity through activation of MAP-kinases JNK and p38 and/or inhibition of BDNF and Arc, essential mediators of hippocampal-dependent memory processes (Barrientos et al., 2015; Lynch, 2015). Furthermore, pro-inflammatory cytokines contribute to negatively influence hippocampal neurogenesis with detrimental consequences for synaptic plasticity phenomena. Therefore, by impairing microglial priming or blocking the excessive brain cytokine response (pharmacologically or through diet and voluntary exercise) it may be possible to effectively attenuate the harmful effects of an immune insult on memory processes, supporting the view that pro-inflammatory cytokines play a pivotal and direct role in inducing long-term memory and cognitive deficits in older individuals (Di Benedetto et al., 2017).

In both MS and EAE, recent studies have shown that inflammatory cytokines are involved in synaptic plasticity alterations. Hippocampal LTP deficits have been detected in EAE mice during the acute phase of the disease, in association with an increased microglial activation, elevated levels of IL-1β, and a selective reduction of NMDA receptors (Di Filippo et al., 2015; Mancini et al., 2017). In MS patients, studies based on transcranial magnetic stimulation (TMS), a non-invasive technique commonly used to detect motor cortex plasticity in humans (Lefaucheur, 2005; Rossini and Rossi, 2007; Ziemann et al., 2008), have shown that synaptic plasticity phenomena are impaired in the progressive forms of the disease, usually diagnosed in people in their 40s (Mori et al., 2013; Stampanoni Bassi et al., 2017). In these patients, that are proned to adaptive immune cell exhaustion form prolonged antigenic exposure, a chronic CNS-intrinsic inflammation and neurodegeneration are prominent, suggesting a concomitant occurrence of cerebral aging and the accumulation of structural brain deterioration (Figure 1). However, a second relevant aspect should be taken in consideration in MS patients. As in other pathological conditions, it is increasingly emerging that synaptic plasticity phenomena, besides having a physiological role in learning and memory processes, drive also important neuroreparative mechanisms (Malenka and Bear, 2004; Pelletier et al., 2009). During MS disease, in the event of an immunological attack, brain damage is eluded by mechanisms of neuroprotection and neurorepair (Weiss et al., 2014; Di Filippo et al., 2015). When these mechanisms eventually fail, an irreversible damage occurs with consequent neuronal denervation. It has been proposed that additional mechanisms, collectively known as brain plasticity, intervene to functionally compensate the deficit of synaptic inputs. The more appropriate synaptic mechanisms underlying brain plasticity and that depend also on the inflammatory milieu are LTP and LTD. In particular, LTP seems to be able to compensate neuronal damage bringing back excitability in synaptic injured neurons. Interestingly, it has been proposed that LTP enhancement might be secondary to alterations of the glutamatergic and GABAergic balance induced by the inflammatory milieu, suggesting that CNS inflammation in MS patients is able to subvert plasticity (Mori et al., 2011, 2012, 2014a; Weiss et al., 2014; Stampanoni Bassi et al., 2017). Furthermore, taking advantage of non-invasive TMS studies, specific brain plasticity alterations have been associated with different disease phenotypes (RR-MS; PP-MS), or phases (remitting, relapsing), indicating that plastic phenomena can modulate disability progression and the clinical manifestation of the disease (Weiss et al., 2014; Stampanoni Bassi et al., 2017).

## Age Is a Risk Factor for MS: Motor Disability, Cognitive Decline and Psychiatric Symptoms

### Motor Disability

Evidence exists that increasing age is a significant factor influencing the clinical course of MS. Indeed, age at onset modifies prognosis and exerts a significant effect on the presenting phenotype of this disease (Confavreux and Vukusic, 2006; Tremlett et al., 2006; Scalfari et al., 2011; Tedeholm et al., 2015). Clinical studies reported that a younger onset age was strongly associated with a slower rate of disability accumulation. As shown in an observational study on 1844 patients, aging negatively affects the prognosis in MS, independently of the type of the initial course of the disease, be it exacerbating-remitting or progressive (Confavreux and Vukusic, 2006). Clinical data from the London Ontario database highlight that onset age of patients with the relapsing form of MS strongly influences the time to conversion to secondary progression; compared to age 20, onset at age 40 and at age 50 doubled and tripled risks of developing SP respectively, compared to age 20 (Scalfari et al., 2011). A clinical study on 500 MS patients, grouped by cutoff in onset age, demonstrated that the severity of disabilities was increased during and after an age band of 30 years–35 years (Ramachandran et al., 2014). In agreement, it has been reported that a younger age (20 years–35 years) is correlated with a longer time to reach disability milestone compared with older patients (36 years–65 years; Trojano et al., 2002), confirming that age at onset predicts the disease course. All together these results are in agreement with previous evidence that neurological relapses have only a limited influence on the risk of entering secondary progressive phase and on the latency of entering progression (Confavreux et al., 2000; Scalfari et al., 2010, 2013).

Furthermore, age at onset strongly predicts the probability of a patient to evolve in a primary progressive form of the disease. Kis et al. (2008) reported that the majority (83%) of MS patients with late-onset MS had a primary progressive disease course, whereas the young-onset MS group (94%) developed a relapsing-remitting form. Accordingly, motor deficits were significantly more frequent in the late-onset MS than in patients with young-onset MS (90% vs. 67%; Kis et al., 2008). Similar results were obtained in a previous study on 957 patients with MS, since age at onset predicted the likelihood of developing a primary progressive form and, for RRMS patients, strongly determined the time to conversion to secondary progression (Stankoff et al., 2007).

The impact of aging on brain pathology was also supported by the observation that median age at the time of assigned disability is substantially similar between patients with different clinical course or symptomatology. Among primary progressive and RR-SP patients, median ages at reaching a given disability score were strikingly similar (Scalfari et al., 2011), suggesting that age at onset has a strong impact on the neurodegenerative component of MS.

### Cognitive Impairment

Together with motor disability, CI is frequently observed in MS patients (with an occurrence estimates between 40% and 65% of MS patients) and tends to progress over time. CI is present since the clinical onset of MS, more frequently in progressive patients compared to RRMS patients (Huijbregts et al., 2004; Ruet et al., 2013; Planche et al., 2016; Matias-Guiu et al., 2017), although heterogeneous results have been reported (Rao, 1991; Potagas et al., 2008).

In a 10-year longitudinal study, Amato et al. (2001) comparing cognitive capacities of 50 MS patients, found that cognitive dysfunction progressed as MS advances and that neurological and cognitive involvement tended to converge during the follow-up. The percentage of cognitive preserved patients decreased from 74% to 51% during 4 years of follow-up, and to 44% after 10-year assessment. In parallel, at the end of the study about 56% MS patients showed mild and moderate CI (Amato et al., 2001).

The relationship between CI, physical disability and age was further investigated in a larger study that compared prevalence and profile of cognitive deficit across 1040 patients with different clinical phenotypes (Ruano et al., 2017), including 167 clinically isolated syndrome, 759 RR, 74 SPMS and 40 PPMS patients. The multivariable analysis showed that higher disability (evaluated by EDSS) and older patient age, rather than clinical subtype and disease duration were the main determinant of CI. Furthermore, the study supported the evidence that the frequency of CI is increased in the progressive forms of the disease. Indeed, SP and PP patients showed approximately two-fold higher prevalence of CI when compared with RR and CIS patients.

The significant correlation with CI and age was reported also in two different Cross-Sectional studies. In the first study, 245 MS patients and 188 healthy control were evaluated using two measures of processing speed (the preliminary word reading and color naming trials of the Stroop) and the analysis were performed grouping participants into five age cohorts (Bodling et al., 2009).

In the second one, regression analyses were performed between age and several cognitive parameters (six outcomes T25FW, 9HPT, PASAT, SDMT, CVLT-II Learning and BVMT-R Learning) in 698 MS patients and 226 healthy subjects (Roy et al., 2017). Surprisingly in both clinical studies, although older MS patients were at higher risk of motor and cognitive disability, as expected, the interaction between MS and healthy control on cognitive test during lifespan was not significantly different, suggesting that age effects on CI are similar across healthy control and MS patients. Despite further longitudinal studies should be performed to determine and clarify the impact of aging on MS cognitive decline, these preliminary studies leave open interesting questions as discussed in the next paragraphs.

### Psychiatric Symptoms

Recent studies have clearly demonstrated that also psychiatric affections are strongly associated with MS pathology. Depression and anxiety are known to be more prevalent among people with MS compared with the general population and individuals with other neurologic conditions (Boeschoten et al., 2017). Recently, several clinical (Imitola et al., 2005; Rossi et al., 2017) and preclinical studies (Haji et al., 2012; Gentile et al., 2016; Mandolesi et al., 2017b) have provided evidence that mood disturbances occur early in MS disease and its mouse model EAE and correlate with peripheral and central inflammation, independently of motor disability. Concerning the correlation between these psychological conditions and aging in MS, conflicting data are emerging. Several cross-sectional studies suggest that younger adults with MS are associated with greater risk for depressive symptoms respect to older patients (Patten et al., 2000, 2003; Chwastiak et al., 2002; Williams et al., 2005; Phillips and Stuifbergen, 2008). Accordingly, Kneebone et al. (2003) observed that older patients (70.6 ± 4.51 years) with MS were less affected by depressive symptoms with respect to younger patients (46.4 ± 8.35 years). Moreover, a significant correlation was found between the younger age at onset and the presence of depression in MS patients (Beiske et al., 2008). Interestingly, Williams et al. (2005) showed that a shorter duration of MS was associated with greater risk for depression. In contrast to these studies, da Silva et al. (2011) found that age was positively associated with depression in MS patients and Mattioli et al. (2011) reported that depressed MS patients were older than those not depressed (43.45 ± 12.15 years vs. 39.96 ± 10.88 years, *p* = 0.02). Finally, several cross-sectional studies on MS patients failed to replicate a relationship between depression and age (Galeazzi et al., 2005; Tsivgoulis et al., 2007; Bamer et al., 2008; Buchanan et al., 2009).

In conclusion, it is still not clear if aging may have an impact on MS psychiatric symptoms and further cross-sectional and longitudinal studies are needed to better investigate this challenging aspect, considering that a complex interplay of variables influences this form of comorbidity in MS (Boeschoten et al., 2017).

Collectively, these data support the hypothesis that in MS the shift from a predominantly inflammatory phase, dominated by clinical relapses, to a predominantly neurodegenerative phase, dominated by irreversible progression of neurological disability (motor and cognitive), may be mainly driven by biological factors related to aging (Lassmann et al., 2012; Friese et al., 2014; Mahad et al., 2015; Ruano et al., 2017; Zeydan and Kantarci, 2018).

## Aging Reduces the Ability to Recover After a Relapse by Affecting Brain Plasticity

Several results concur with the hypothesis that the capacity of the brain to manage MS pathology depends on the ability to recover after a relapse. Of note, the ability to recover was strongly correlated to reserve of brain plasticity, which are diminished in older patients. A poor recovery of early relapses is indeed associated with an earlier progression of the pathology (Kalincik et al., 2014; Novotna et al., 2015). On the other hand, relapses with a higher impact and poorer recovery in MS patients were positively correlated with age (Kalincik et al., 2014) as well as a reduced ability to recover from initial relapse significantly declined with age (Cossburn et al., 2012). Aging has been indeed related to a decreased capability of functional reorganization and plasticity in MS, likely due to an interaction between cerebral aging and the accumulation of structural brain damage (Schoonheim et al., 2010).

It is now well recognized that MS-associated pathological processes progressively modify brain networks essential for functional domains such as sensorimotor function (Rocca et al., 2005; Tomassini et al., 2012), vision (Jenkins et al., 2010) and cognition (Rocca et al., 2015), by activating adaptive or maladaptive mechanisms. A form of adaptive plasticity can be considered the functional reorganization observed in the brain of MS patients in association to the easily performance of simple tasks; by means of this compensatory mechanism, more complex brain systems are recruited relatively to normal subjects (Pelletier et al., 2009). On the other hand, forms of maladaptive plasticity might also occur, causing functional changes directly linked to disability (Reddy et al., 2002). Functional MRI has contributed notably to improve our understanding of the mechanisms associated with preserved function in MS (Mainero et al., 2006; Filippi and Rocca, 2009; Rocca et al., 2010b, 2005; Enzinger et al., 2016). Thanks to these studies, it has been proposed that (Filippi and Rocca, 2009; Rocca et al., 2015) an increased involvement of the cortical networks might help at containing the functional impact of MS-related damage (Rocca et al., 2002a) and that changes in the organization of cortical areas involved in motor or cognitive tasks at different stages of the disease (Reddy et al., 2000a,b, 2002; Filippi et al., 2002a,b; Pantano et al., 2002; Rocca et al., 2002a,b, 2003a,b; Enzinger et al., 2016), might in part explain the discrepancy between brain injury and clinical disability. The progressive weakeaning of patterns of activation might account for progressive disability or CI in symptomatic MS patients, when compared to controls (Rocca et al., 2002b, 2010a; Ciccarelli et al., 2006) or asymptomatic MS patients (Penner et al., 2003; Mainero et al., 2006; Rocca et al., 2010a).

A deeper understanding of causal and functional relationships were achieved by neurophysiological techniques, predominantly by TMS (Zeller and Classen, 2014). Notably, accumulating evidence obtained through this technique have highlighted the ability of the brain to express LTP-like changes as a determinant factor to counteract disability progression in MS (Weiss et al., 2014; Stampanoni Bassi et al., 2017). Recently, it has been shown that a predictor of disease recovery from relapses is LTP induced by paired associative stimulation (Mori et al., 2014b). Furthermore, it has been observed that LTP induction in the primary motor cortex of patients in the early relapsing-remitting phases of the disease is possible and even enhanced. Conversely, LTP is absent in patients with a progressive form of MS (Mori et al., 2013; Weiss et al., 2014). Thus, it is conceivable that the capability of brain networks to adjust themselves in a plastic manner, allows a patient to accomplish with the focal and diffuse brain damage associated with the disease in the first years after MS onset. On the contrary, the disease likely enters in its more disabling progressive phase when the plastic reserve is exhausted. Accordingly, a study conducted on a cohort of RRMS patients with CI and on a group of cognitively preserved RRMS patients (Mori et al., 2011) revealed that LTP response was present only in the cognitively preserved group. Synaptic plasticity induction by PAS was also explored in RRMS and SPMS patients with stable clinical conditions in the last 3 months (Zeller et al., 2010), resulting in a comparable LTP amplitude between patients and gender, age-matched healthy controls. These observations suggest that alterations of synaptic plasticity emerge at the time of a relapse, as explored by other groups using paired associative stimulation (Mori et al., 2014b) or intermittent-theta burst stimulation (Mori et al., 2011).

Of note, LTP responses are variable among individuals and tend to decline with aging (Müller-Dahlhaus et al., 2008; Freitas et al., 2011). Accordingly, MS patients in their forties suffer from progressive forms during which LTP seems to be impaired. Current hypothesis to explain this inter-individual variability points to a combination of disease, age and likely genetic background (Pascual-Leone et al., 2011; Rossi et al., 2013) but further studies are necessary to clarify how population-level variability in LTP induction is related to MS disease phenotype and expression.

## Does Aging Differentially Affect Motor and Cognitive Function in MS Patients?

We have summarized several data concurring with the hypothesis that the interaction between cerebral aging and overload of structural brain injury decreases plasticity and capability of functional reorganization in MS (Figure 1). However, unexpectedly, recent studies suggest that aging processes differentially affect motor and cognitive performances in patients with MS (Bodling et al., 2009; Roy et al., 2017). It seems that older MS patients are more susceptible to motor disability, and less to cognitive dysfunction. Although longitudinal studies, which should include both healthy controls and different MS subgroups, are demanded to better clarify this aspect, some speculations on this topic might be raised. Among different hypotheses, it has been suggested that in older MS patients, shorter axonal fibers are more prone to resilience and compensation than single long fibers that intervene in motor function. It might be also possible that a prominent and progressive damage of the spinal cord would also have a greater consequence on motor function over time. Furthermore, it is possible that a considerable effect on cognitive rather than motor abilities is played by the influence of cognitive reserve (CR) and personality traits (Sumowski et al., 2013; Roy et al., 2016). CR is indeed emerging as a second mechanism that might contribute at limiting cognitive deficits in MS patients (Rocca et al., 2018). Of note, the theory of CR emerged from aging and dementia studies to justify the inter-individual differences in the ability of managing with brain damage and the consequent cognitive deficits by means of pre-existing compensatory mechanisms (Stern, 2002). According to the CR theory, recent studies showed that intellectual enrichment (such as educational level, vocabulary knowledge, employment status), protects MS patients against the negative effect of disease-related damage on cognitive performance (Sumowski et al., 2009; Benedict et al., 2010; Ghaffar et al., 2012; Amato et al., 2013; Pinter et al., 2014; Rocca et al., 2018). In particular, it has been suggested that CR may have a protective role in preserving cognitive functions, mitigating the effect of structural damage on cognitive performance. However, in recent 2-years MS longitudinal study emerged that the CR protective role may diminish with disease progression.

Notably, it should be also taken in consideration that brain compensatory and adaptive mechanisms might depend on a genetic background. In this regard, recent evidence suggests that genetic determinants influence inter-individual differences in both MS severity and synaptic excitability (Rossi et al., 2013). In particular, four single nucleotide polymorphism (SNPs: rs4880213, rs6293, rs1805247, rs7301328) of the NMDAR genes were evaluated to search for associations with both synaptic excitability and disease outcome in RRMS and PPMS patients, considering that LTP is dependent on the synaptic activation of the glutamate NMDA receptor. This study showed that the specific T allele of the rs4880213 SNP of the NR1 subunit was correlated to an increase of neuronal excitability measured by means of paired TMS protocol. Furthermore, the same SNP was associated with better compensation of brain damage in RR-MS but with higher severity of PP-MS. Accordingly, these results supported the idea that potentiation of glutamatergic NMDAR-dependent synaptic transmission has a role in both adaptive plasticity and excitoxicity in MS patients. More interestingly, enhanced NMDAR function was found to preserve cognitive abilities in that cohort of patients, thus confirming the role played by NMDARs and NMDAR-dependent synaptic plasticity during learning and memory processes (Collingridge and Bliss, 1995; Nicoll and Malenka, 1999; Bennett, 2000; Cooke and Bliss, 2005, 2006). Notably, both RR-MS and PP-MS carrying the T allele of rs4880213 SNP showed a better performance at the Paced Auditory Serial Addition Test and the Symbol Digit Modalities Test, indicating residual NMDAR-dependent plasticity also in PP-MS. The above mentioned clinical tests explore cognitive domains frequently altered in MS, and impaired NMDAR-dependent synaptic plasticity in MS patients has been associated with poor performances at both tests (Mori et al., 2011). This evidence supports the idea that genetic determinants (SNPs) in combination with age and disease, might explain inter-individual variability of LTP responses (Pascual-Leone et al., 2011).

Although both genetic variability and CR could in part contribute to explain a potential less impact of age on cognitive decline, collectively these data clearly indicate that compensatory mechanisms of the brain can be potentially activated and responsive to therapeutic interventions even in PPMS patients (Feinstein et al., 2015). In this regard, despite some controversial results, it seems that increased physical activity is promising for cognitive benefits in MS (Dalgas and Stenager, 2012; Motl and Pilutti, 2012; Beier et al., 2014; Briken et al., 2014; Sandroff et al., 2014, 2016a; Motl et al., 2017). Indeed, in a randomized controlled trial of 42 patients with progressive MS (31 SPMS and 11 PPMS) with moderate physical disability (EDSS score 4–6), patients committed to several forms of exercise, rather than a wait list group, showed a significant amelioration in aerobic fitness and several secondary outcome measures, including cognitive performance (Briken et al., 2014). This study highlighted for the first time that physical activity specifically target cognition in patients with progressive MS but further studies are needed to identify the best type of exercise for cognitive recovery (Sandroff et al., 2016b; Motl et al., 2017; Ontaneda et al., 2017). Of note, in the aging context, it is emerging that exercise, as well as pharmacological treatments, may effectively block the detrimental effects of an immune challenge by preventing microglial priming or blocking the overstated brain cytokine response. This evidence not only suggests that these interventions may be useful therapeutic treatments, but also supports the view that a dysregulation of the fine interplay between the immune and nervous systems can have profound impacts on long-term plasticity and cognitive function in older individuals, as occurs in MS (Di Benedetto et al., 2017).

## Conclusion

**Figure 2:**
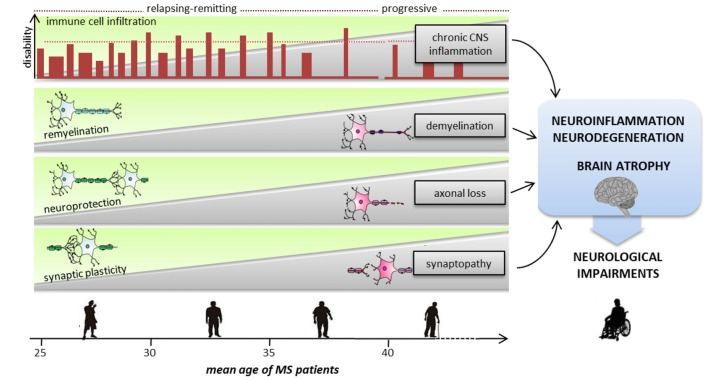
Sequence of pathological and reparative events occurring during MS disease course, leading to severe neurological impairments. With advancing aging, neurodegenerative and neuroinflammatory processes are exacerbated causing severe cognitive and motor deficits. Inflammation is present at all stages of the disease, starting from an early phase dominated by peripheral immune cells invasion into the CNS to a progressive and chronic CNS inflammation. Since the early phase of the relapsing/remitting forms demyelination, synaptopathy and axonal damage occur causing progressive neurodegeneration. To avoid brain damage and to functionally compensate for eventual deficits of synaptic inputs, mechanisms of neuroprotection, remyelination and synaptic plasticity intervene. The transition from relapsing-remitting course MS (RRMS) to secondary progressive MS (SPMS) is likely to be the point at which the compensatory brain plasticity reserve bypassing of neuronal injury is exhausted. Usually after the fifth decade, regardless of the preceding disease course and severity, the inevitability and the rate of neurological decline are highly consistent.

MS patients are indubitably exposed to the biological consequences of age, but the precise contribution of aging-related factors to MS neurological disability and the underlying molecular and cellular mechanisms are still unclear. Recent experimental and clinical investigations in the field of both aging and MS have shed a light onto common neuroinflammatory pathological mechanisms that are likely prematurely activated or exacerbated in MS patients, influencing the expression of the MS disease course. Here, we emphasize how a dysregulated synergy between the immune and nervous systems in the course of MS disease causes inflammation-dependent synaptic dysfunction, plasticity and degeneration with important implications on motor and cognitive functions. Since the early phase of MS, inflammatory dependent synaptopathy and synaptic plasticity perturbations seem to occur even independently of demyelination. As MS disease progresses, besides a decrease in remyelinating processes, brain repair and plasticity become exhausted while excitotoxic and neurodegenerative processes are exacerbated in association to a chronic CNS inflammation, supporting a late contribution of aging processes (Figure 2). Accordingly, cognitive dysfunction is now recognized to be an early symptoms of MS disease and for this reason it is included in diagnostic, follow-up and therapeutic evaluations (Nasios et al., 2018).

Finally, we highlighted unexpected results obtained from clinical studies conducted on MS patients and healthy controls, revealing a milder effect of aging on MS cognitive function relative to motor disability. Although further investigations are necessary to clarify this aspect, it would be interesting to investigate also which compensatory mechanisms in MS brain are specifically involved. Considering the emerging view of a bidirectional communication between the nervous system and immune system, in which neuroinflammatory processes can be adaptive and beneficial (Schwartz and Deczkowska, 2016), it may be suggested that in the aging MS brains, a vicious interaction between the compromised immune and neuronal systems culminates in a protective and adaptive mechanisms of the neuro-immune system.

In conclusion, a more precise comprehension of the temporal (with age) and spatial distribution and activity of immune players in MS brain in association with specific stages of disease, genetic determinants and brain plasticity reserve is essential to determine the deep nature of the pathology, and thus the ideal strategy required to treat it.

## Author Contributions

AM, DC and GM conceived and designed the manuscript. All the other authors made significant contributions, reviewed and approved the manuscript.

## Conflict of Interest Statement

The authors declare that the research was conducted in the absence of any commercial or financial relationships that could be construed as a potential conflict of interest.

## Abbreviations

AMPA: α-amino-3-hydroxy-5-methyl-4-isoxazole propionic acid
BDNF: brain derived neurotrophic factor
CI: cognitive impairment
CNS: central nervous system
CR: cognitive reserve
EAE: experimental autoimmune encephalomyelitis
GABA: γ-aminobutyric acid
IGF-1: insuline-like growth factor
IL-17: interleukin-17
IL-1β: Interleukin-1β
IL-6: Interleukin-6
ISC: immunosenescence
IFNγ: interferon-γ
LTD: long-term depression
LTP: long-term potentiation
MS: multiple sclerosis
NMDA: N-methyl-D-aspartate
PPMS: primary progressive MS
RNS: reactive nitrogen species
ROS: reactive oxygen species
RRMS: relapsing–remitting course
SNP: single nucleotide polymorphism
SPMS: secondary progressive MS
TMS: transcranial magnetic stimulation
TNF: tumor necrosis factor.
